# Reactivity to Novel Autoantigens in Patients with Coexisting Central Nervous System Demyelinating Disease and Autoimmune Thyroid Disease

**DOI:** 10.3389/fimmu.2017.00514

**Published:** 2017-05-08

**Authors:** Judith M. Greer, Simon Broadley, Michael P. Pender

**Affiliations:** ^1^Centre for Clinical Research, The University of Queensland, Brisbane, QLD, Australia; ^2^School of Medicine, Griffith University, Southport, QLD, Australia; ^3^Department of Neurology, Gold Coast University Hospital, Southport, QLD, Australia; ^4^Faculty of Medicine, The University of Queensland, Brisbane, QLD, Australia; ^5^Department of Neurology, Royal Brisbane and Women’s Hospital, Brisbane, QLD, Australia

**Keywords:** multiple sclerosis, neuromyelitis optica spectrum disorder, autoimmune thyroid disease, calcitonin gene-related peptide, LGR4, T cells, autoantibodies, autoantigens

## Abstract

Several lines of evidence suggest a definite and unique link between CNS demyelinating diseases and autoimmune thyroid disease (AITD). The aim of the current study was to systematically compare the clinical and laboratory features of patients with coexistent AITD and CNS demyelinating disease with those of patients with just CNS demyelinating disease. Forty-four patients with coexisting CNS demyelinating disease and AITD were identified and their clinical and radiological features were recorded. Blood and DNA were collected and tested for HLA type and for the response of T cells and antibodies to a variety of antigens. Patients with multiple sclerosis (MS) without AITD and healthy individuals were included as controls. Patients with coexisting AITD and CNS demyelinating disease were almost exclusively female (43/44) and had prominent spinal cord involvement as the main neurological finding. The HLA molecules carried by individuals with CNS demyelinating disease and AITD differed from both other MS patients and healthy individuals. Furthermore, patients with both CNS disease and AITD showed less T cell reactivity than patients with MS alone to myelin proteolipid protein, but, compared to other groups, showed elevated levels of T cell reactivity to the calcitonin gene-related peptide, which is present in both the CNS and the thyroid, and elevated levels of T cell and antibody to the leucine-rich repeat-containing G-protein coupled receptor 4 (LGR4), a molecule that is expressed in the brainstem and spinal cord, and which is a homolog of the thyroid-stimulating hormone receptor. We suggest that reactivity of autoreactive immune cells in these patients against antigens present in both the thyroid and the spinal cord is a potential mechanism underlying the pattern of lesion development in the CNS in patients with coexisting AITD and MS and might indicate a novel mechanism of disease pathogenesis in these patients.

## Introduction

We have had a long-term interest in the question of why some patients with autoimmune CNS demyelinating disease tend to develop lesions in relatively restricted parts of the CNS. In some patients, lesion distribution appears to correlate closely to the HLA type of the patients and the dominant myelin antigen-specific T cell reactivity restricted by those HLA types (1, 2). In other cases, we have noted that patients who have another coexisting autoimmune disease often tend to have a similar lesion distribution, HLA restriction, and T cell myelin antigen reactivity pattern when compared to other patients who have the same combination of autoimmune diseases. For example, we have shown that patients with a combination of multiple sclerosis (MS) and psoriasis tend to have prominent involvement of the brainstem and cerebellum lesions and to carry HLA-DRB1*07 (3) and have elevated levels of T cell reactivity against the 184–210 region of myelin proteolipid protein (PLP) (unpublished data).

Neuromyelitis optica (NMO) is another example of restricted lesion distribution in CNS demyelinating diseases, being originally characterized primarily by the presence of optic nerve and longitudinally extensive spinal cord lesions and the presence of aquaporin 4 (AQP4) autoantibodies. More recently, NMO spectrum disorders (NMOSD) has become the favored terminology for this syndrome, as it is now recognized that some patients have variant lesion distribution, with the presence of either autoantibodies against AQP4 or myelin oligodendrocyte glycoprotein (MOG). Interestingly, it has been reported that there is an increased incidence of non-organ-specific autoimmunity in NMOSD (4), and the coexistence of systemic lupus erythematosus (SLE) or Sjögren’s syndrome in AQP4 autoantibody positive patients with NMOSD actually strengthens confidence in the NMOSD diagnosis (5).

In both MS and NMOSD, there have been reports of increased prevalence of thyroid dysfunction and anti-thyroid antibodies (6–10). We have observed that MS patients who have coexisting autoimmune thyroid disease (AITD) tend to have more extensive spinal cord involvement than other MS patients, irrespective of whether the MS or the thyroid disease develops first (11). Links between disease affecting the corticospinal tract and thyroid disease have been recognized for many years. In 1888, Charcot described paraplegia-like symptoms in a patient with severe hyperthyroidism (12). Hyperthyroidism has been reported as a contributing factor in corticospinal tract malfunction (13) and posterolateral myelopathy (14), and encephalopathy associated with hypothyroidism (Hashimoto encephalopathy) has been recognized for many years (15). In addition, increased IgG, myelin basic protein and activated helper T cells have been found in the cerebrospinal fluid of acute necrotizing myelopathy associated with thyroid cancer, suggesting that immune-mediated demyelination may be occurring in this condition (16).

There could be several explanations for the links between autoimmune CNS demyelinating disease and AITD. First, the HLA type of patients might predispose them to development of both AITD and CNS demyelinating disease, by allowing presentation of pathogenic epitopes specific for each of these disorders. The two main types of AITD, Graves’ disease (hyperthyroidism) and Hashimoto’s thyroiditis (hypothyroidism), have both been linked primarily to carriage of the HLA-DRB1*03-DQB1*02-DQA1*0501 (DR3) genotype in Caucasians (17). MS is generally thought of as involving linkage to HLA-DRB1*15:01; however, around 40% of MS patients are negative for DRB1*15:01 (1), and HLA-DRB1*03 has also been reported to confer an increased risk for development of MS (18). Interestingly, studies on NMOSD patients from Caucasian, Afro-Caribbean, and Indian populations have all found an association with HLA-DRB1*03 (19–21). Second, there may be cross-reactivity between antigens present in the CNS and in the thyroid, either through expression of the identical antigens in both organs or through expression of related proteins with conserved epitope domains in the different organs. Other possibilities include altered thyroid function inducing changes in the CNS, including the induction of neoantigens; however, our observation that patients with coexisting CNS and thyroid disease have similar patterns of lesion distribution, irrespective of whether they initially had CNS or thyroid disease, suggests that this last mechanism is less likely. Finally, molecular mimicry between various microbes and self-antigens has been postulated to underpin development of autoimmunity, and it is of interest to note that MS, NMOSD, and AITD have all been linked to infection with microbes, such as hepatitis C virus and *Helicobacter pylori*, suggesting the possibility that such infections could induce autoimmune diseases targeting more than one organ (22–26).

The aim of the current study was to investigate HLA molecules and T cell and autoantibody reactivity to CNS and thyroid antigens in patients with coexisting CNS demyelinating disease and AITD. We show that such patients differ in these respects from healthy individuals and MS patients without AITD.

## Materials and Methods

### Patients and Controls

This study was approved by the Royal Brisbane and Women’s Hospital Human Research Ethics Committee, The University of Queensland Medical Research Ethics Committee, and Griffith University Human Research Ethics Committee. Up to 20 mL of blood was obtained from patients with coexisting autoimmune CNS demyelinating disease and AITD, patients with MS alone, and healthy individuals who had no history of AITD. Written informed consent was obtained prior to blood collection. Where sufficient blood was available, 5 mL was used as a source of serum, 2 mL was used to extract genomic DNA, and the remainder was used for T cell assays. Only 3–5 mL of blood was available from four patients with autoimmune CNS demyelinating disease and AITD, in which case plasma was used rather than serum, and T cell assays were tested only for reactivity to one of the antigens of interest, calcitonin gene-related peptide (CGRP—see below for more detail on antigens used). Blood was not available from 11 of the patients with coexisting AITD and MS. Patient and control demographics for individuals from whom blood was collected are shown in Table 1.

**Table 1:** **Demographics of patients and controls whose blood was used in this study**.

Group	*N*	Age at time of blood collection (mean ± SE)	M:F	CNS disease type				Disease duration (mean ± SE)	EDSS (mean ± SE)
				RR-MS	SP-MS	PP-MS	RR-NMO		
CNS demyelinating disease + autoimmune thyroid disease	33	50.9 ± 1.8	1:32	12	11	6	4	13.1 ± 1.8	5.1 ± 0.4
Hypo	19	53.7 ± 2.2	1:18	5	6	5	3	15.5 ± 2.3	5.4 ± 0.4
Hyper	14	47.1 ± 3.0	0:14	7	5	1	1	10.1 ± 2.3	4.6 ± 0.8
Other multiple sclerosis (MS) (T cell assays)	20	46.0 ± 3.0	4:16	12	5	3	0	10.6 ± 2.4	4.3 ± 0.7
Other MS (LGR4 and thyroid-stimulating hormone receptor serum assays)	13	41.1 ± 3.0	3:10	10	1	2	0	10.1 ± 1.9	3.3 ± 0.5
Other MS (calcitonin gene-related peptide serum assays)	14	39.0 ± 2.5	2:12	9	2	3	0	7.2 ± 2.5	4.0 ± 0.7
Healthy controls (HC) (T cell assays)	20	41.4 ± 2.5	4:16	NA	NA	NA	NA	NA	NA
HC (serum assays)	11	36.2 ± 3.1	1:10	NA	NA	NA	NA	NA	NA

*M:F, number of males:number of females; RR-MS, relapsing-remitting multiple sclerosis; SP-MS, secondary progressive MS; PP-MS, primary progressive MS; RR-NMO, relapsing-remitting NMO; EDSS, Expanded Disability Status Scale score; NA, not applicable*.

### HLA Typing

Genomic DNA was prepared using NucleoSpin Blood DNA extraction kits (Macherey-Nagel, Düren, Germany) as previously described (2). Dynal low and high resolution SSP kits (Dynal Biotech, Thermo Fisher, Australia) were used to type for HLA-DR, -DQA, -DQB, and -DP alleles, following the manufacturer’s recommended protocols. Results for the patients with coexisting CNS demyelinating disease and AITD are reported to the four digit level, when it was able to be determined. For comparison with larger groups of MS patients and healthy controls (HC), alleles were grouped at the two digit level.

### Peptides and Antigens

For T cell assays, peptides from PLP, the thyroid-stimulating hormone receptor (TSHR), LGR4 (a homolog of the TSHR), and the α and β types of CGRP were used to stimulate T cells. The PLP peptides used (PLP_184–199_ + PLP_190–209_) were overlapping peptides against which we have previously reported elevated levels of T cell reactivity in approximately 40% of MS patients (2). TSHR and LGR4 are members of the same LGR family and have some regions of sequence homology (27). The three peptides chosen from each of these molecules are from the extracellular portion of the protein in each instance. CGRP is a neuropeptide that has been reported to be expressed in both the thyroid and the spinal cord (28). The sequences of peptides used are given in Table 2. α-CGRP and β-CGRP were purchased from Bachem (Bubendorf, Switzerland). The other peptides were synthesized by Mimotopes (Melbourne, VIC, Australia). Because the PLP peptides are moderately hydrophobic they were dissolved in 0.2 M acetic acid as 5 mg/mL stock solutions. Stock solutions of the other antigens were made in water. The peptides were diluted in tissue culture medium immediately prior to use. For T cell assays, peptides were used in four pools: CGRP (α-CGRP + β-CGRP), PLP (PLP_184–199_ + PLP_190–209_), LGR4 (pool of the three LGR4 peptides), and TSHR (pool of the three TSHR peptides). Concanavalin A (Con A) was used as a positive control in all T cell assays.

**Table 2:** **Sequences of peptides used in this study**.

Peptide designation	Sequence
PLP_184–199_	QSIAFPSKTSASIGSL
PLP_190–209_	SKTSASIGSLCADARMYGVL
CGRPα	ACDTATCVTHRLAGLLSRSGGVVKNNFVPTNVGSKAF
CGRPβ	ACNTATCVTHRLAGLLSRSGGMVKSNFVPTNVGSKAF
LGR4_38–51_^a^	DRRVDCSGKGLTAV
LGR4_529–542_^a^	FKPCEYLLGSWMIR
LGR4_609–623_^a^	GIWWETGSGCKVAGS
TSHR_36–49_^b^	DFRVTCKDIQRIPS
TSHR_405–418_^b^	FNPCEDIMGYKFLR
TSHR_485–499_^b^	AIDWQTGPGCNTAGF

*^a^Accession number for LGR4 sequence is Q9BXB1*.

*^b^Accession number for thyroid-stimulating hormone receptor sequence is P16473*.

### Assessment of T Cell Proliferation

Blood was centrifuged through Ficoll and the peripheral blood mononuclear cells (PBMC) were collected at the interface, washed, and used fresh in T cell assays. Assays testing for reactivity to CGRP were done using a ^3^H-thymidine uptake assay, as previously described (2). In brief, 1.5 × 10^5^ fresh PBMC/well were cultured in U-bottom 96-well plates (Nunc) in the presence or absence of CGRPα + CGRPβ (at concentrations ranging from 1 to 50 µg/mL) or Con A (2 µg/mL) for 6 days, with [^3^H]thymidine being added during the final 18 h. Cells were then harvested onto glass-fiber mats, and the cpm determined in a Betaplate counter (Beckman Coulter). The cell division index (CDI) was determined by the formula: CDI = (Mean cpm of peptide-containing wells)/(Mean cpm of control wells without peptide). The mean CDI reported is the maximum value over the different antigen dilutions. For the other antigens, reactivity was tested using CFSE assays, as we have previously described (2). Briefly, PBMC were labeled with 2 nM CFSE (Invitrogen), as previously described (29) and incubated with peptide pools (tested at 10 and 25 µg/mL concentrations of each peptide) for 10 days in phenol red-free X-vivo 15 serum-free medium (Lonza). Cells were then washed, stained with PerCP-labeled anti-CD4 or anti-CD8 antibodies (BD Biosciences), and analyzed by flow cytometry, with gating on the lymphocyte population. The number of cells with reduced CFSE staining in the CD4^+^ or CD8^+^ group divided by the total number of CD4^+^ or CD8^+^ cells was used to determine the percentage of cells dividing in response to antigen. The CDI was calculated as the percentage of cells dividing in response to antigen/percentage of cells dividing without antigen. The highest level of proliferation is reported in the results, regardless of the concentration of antigen or whether the responding cells were CD4^+^ or CD8^+^ (the CD4^+^ response was the highest is all expect one assay), in order to keep the results compatible with the assays for CGRP, where the type of responding cell is not determined.

### Detection of CGRP Antibodies

ELISA was used to test for the presence of antibodies against CGRP. Plates (Nunc Maxisorb) were coated with a mixture of α-CGRP and β-CGRP (each at 0.5 μg/well) + bovine serum albumin (BSA) (2.5μg/well) or with BSA (2.5μg/well) alone (control wells) in 0.2 M bicarbonate buffer. Plates were blocked with 2% skimmed milk in PBS-Tween 20, 100 µL of each diluted serum or plasma sample (1/40 dilution) was added in triplicate to control wells and wells containing CGRP, and the plates incubated overnight at room temperature in a humidified chamber. After four washes, 100 µL of alkaline phosphatase-labeled anti-human polyvalent Igs (G + A + M) (Sigma-Aldrich) was added to each well and plates were incubated for 2 h at room temperature in a humidified chamber. Plates were washed again and 200 µL of *p*-nitrophenyl phosphate substrate (Sigma-Aldrich) was added to each well. The reaction was stopped after 15 min by addition of 25 µL 3N NaOH, and the absorbance read at 405 in a plate reader (Paradigm). The CGRP-specific response was determined by subtracting the mean absorbance value for the wells coated with BSA alone from that of wells coated with BSA + CGRP.

### Cell-Based Assays to Test for Antibodies against AQP4, MOG, TSHR, and LGR4

Anti-AQP4 and anti-MOG antibodies were detected by fixed cell-based assay using a commercially available kit as per the manufacturer’s instructions (EUROIMMUN, Germany). The presence of antibodies specific for TSHR and for LGR4 was assessed using Cos7 cells transduced with lentiviral particles expressing full length human TSHR or LGR4 (under the control of a cytomegalovirus promoter) together with a GFP marker (to identify transduced cells) and puromycin resistance gene (for selection of transduced cells) were obtained from Applied Biological Materials Inc. (Richmond, BC, Canada). COS-7 cells were grown to 75% confluency and then transduced using polybrene-assisted uptake of particles, using the manufacturer’s recommended protocol. Successfully transduced cells were selected in media containing 20 µg/mL puromycin. The transduced cells or non-transduced Cos7 cells (as controls) were plated in 16-well chamber slides. Once cells were confluent, the slides were washed, fixed in freshly prepared 4% paraformaldehyde, and then stored in PBS with 0.01% azide at 4°C until required. Sera/plasma was added to wells at 1:20 dilution (1 h at 4°C). Each sample was tested on at least two wells of each cell line. Each slide contained control wells that had either no primary antibody or no primary and no secondary antibody added. After 3× 5 min washes in PBS-T, HRP-labeled anti-human Ig (G + A + M) was added to wells for 1 h at 4°C. Slides were washed again, and then nickel-enhanced DAB substrate (Sigma) was added for 5 min. Cellular DAB staining was assessed in a blinded fashion for each well.

### Statistical Analyses

GraphPad Prism 7.0 was used for analysis. For comparisons of frequencies, contingency tables were analyzed using a two-way Fisher’s exact test. Where correction for multiple comparisons was applied, that is noted in the text. Where a comparison among three or more groups was to be made, data were first assessed to determine if they were normally distributed. If they were, ANOVA with Bonferroni correction for multiple comparisons was used to compare groups. If the data were not normally distributed, the Kruskal–Wallis test with Dunn’s multiple comparisons test was used. *p* < 0.05 was considered to be significant throughout.

## Results

### Features of Patients with Coexisting CNS Demyelinating Disease and AITD

We identified 44 patients with CNS demyelinating disease who had also been diagnosed with coexisting AITD. Of these 44 patients, all were of Caucasian background and only 1 (2.3%) was male. Four patients (10.8%, all female) met diagnostic criteria for NMOSD (5) and were positive for anti-AQP4 antibodies using a cell-based assay. All other patients (except case #227) met the 2005 and/or 2010 Revised McDonald criteria for MS (30, 31) and were negative for anti-AQP4 and anti-MOG autoantibodies using cell-based assays. Of the 40 MS patients with coexisting AITD, the distribution of disease course (50% relapsing-remitting MS, 33% secondary progressive MS, and 17.5% primary progressive MS) is typical for MS. Also in this group, symptoms of AITD started several months after commencement of treatment with IFN-β in four female patients, as we have previously reported in two of these patients (32); these patients were excluded from some of the analyses, as outlined in subsequent sections. None of the patients in the study were treated with alemtuzumab (which has also been linked to subsequent development of AITD).

The clinical characteristics of patients with CNS demyelinating disease and AITD are presented in Table 3. The majority of patients [25/40 (62.5%) in the MS group and 3/4 (75%) in the NMOSD group] had hypothyroidism. Of the 28 hypothyroid patients, 20 had overt hypothyroidism, 4 had subclinical disease, and this information was not recorded for the other four patients. Of the patients with hyperthyroidism, all but one had been treated with radioactive iodine or by thyroidectomy, and were taking thyroxine at the time of blood collection. The hyperthyroid patient who had not had such treatment was in remission at the time of blood collection. None of the hyperthyroid patients had ophthalmopathy. Excluding the patients who developed AITD following commencement of IFN-β treatment, the onset of AITD preceded that of CNS demyelinating disease in 17 patients, developed at the same time in 3 cases (including two of the patients with NMOSD), followed the onset of CNS demyelinating disease in 17 cases, and was unknown for 3 cases. Interestingly, however, even in this small number of patients there was a significant difference (*p* < 0.05) in the proportions of hypothyroid vs hyperthyroid patients who developed AITD before CNS demyelinating disease (Table 4). Hyperthyroidism preceded the onset of CNS demyelinating disease in the majority of cases (66.7%), whereas hypothyroidism occurred prior to development of CNS demyelinating disease in only 31.8% of cases.

**Table 3:** **Clinical characterististics of patients with multiple sclerosis (MS) and autoimmune thyroid disease (AITD)**.

ID No	Diagnosis	Cerebrospinal fluid Oligo IgG	Age MS onset	Age AITD onset	Thyroid type	Other autoimmune disease	Family history of autoimmunity	Major site of clinical involvement at attack						
								1	2	3	4	5	6	7
133	RR-MS	NT	43	42	Hyper	None	Yes	SC	SC	SC	BS Cb	SC		
200	RR-MS	NT	32	16	Hypo	None	Yes	SC	ON	SC	SC	SC ON		
269	RR-MS	NT	32	28	Hyper	None	Yes	SC	SC	SC				
271	RR-MS	Yes	29	26	Hyper	None	Yes	SC	SC Cb	BS	SC			
274	RR-MS	NT	35	<22	Hyper	ANA 1:40	Adopted	ON SC	SC	SC	SC			
291	RR-MS	NT	23	51	Hypo	None	No	ON	ON	SC				
299	RR-MS	Yes	57	51	Hyper	None	Yes	BS SC	BS	SC	Cerebral	SC		
316	RR-MS	Yes	41	38	Hypo	None	Yes	SC	SC	SC	SC	SC		
332	RR-MS	No	43	50	Hypo	None	Yes	SC	BS SC					
393	RR-MS	NT	55	35	Hyper	None	Yes	SC	SC					
493	RR-MS	NT	33	42 (after IFNβ)	Hyper	Crohn’s	No	SC	SC	SC	BS SC			
582	RR-MS	NT	30	38 (after IFNβ)	Hypo	None	Yes	Cerebrum	SC	SC	SC			
609	RR-MS	Yes	35	30	Hyper	None	Yes	ON	SC					
610	RR-MS	Yes	17	44 (after IFNβ)	Hypo	None	Not known	ON	BS	SC	SC	SC		
611	RR-MS	NT	43	~50	Hyper	None	Yes	ON Cb	BS	SC	BS			
612	RR-MS	NT	25	37	Hypo	None	No	ON	BS	SC	SC			
613	RR-MS	Yes	40	Not known	Hypo	None	Yes	BS	BS	BS	BS	BS	BS	BS
615	RR-MS	NT	24	44	Hypo	None	Yes	SC	BS					
616	RR-MS	Yes	43	Not known	Hypo	None	Not known	SC	SC	SC	SC	ON		
617	RR-MS	NT	35	36	Hypo	None	Yes	BS	SC	SC				
31	SP-MS	No	34	55	Hypo	Positive lupus anticoagulant	Yes	SC	BS	SC	SC	BS	BS ON	SC
43	SP-MS	Yes	26	48	Hypo	None	Yes	SC	Progressive SC					
145	SP-MS	NT	40	65	Hyper	Autoimmune hepatitis	Yes	SC	Cerebrum, SC and ON in secondary progression					
171	SP-MS	NT	26	45	Hypo	None	Yes	SC	SC BS	SC ON	Further attacks of unclear localization			
182	SP-MS	Yes	23	29	Hyper	None	Yes	BS	ON	Progressive SC				
261	SP-MS	Yes	39	16	Hypo	Possible uveitis	Yes	SC	SC	SC	SC	SC ON	SC	SC
317	SP-MS	Yes	37	49	Hypo	Psoriasis	Yes	BS	SC	Progressive SC				
370	SP-MS	NT	40	19	Hyper	None	Yes	ON	SC	Progressive SC				
374	SP-MS	No	36	Not known	Hypo	None	Yes	BS	SC	SC	Progressive SC			
492	SP-MS	Yes	30	34	Hypo	Psoriasis	No	BS	SC	SC	ON		Progressive SC	
494	SP-MS	NT	39	47	Hyper	None	No	ON	Progressive SC					
593	SP-MS	Yes	35	49	Hyper	None	Not known	BS	SC	SC				
608	SP-MS	Yes	23	37 (after IFNβ)	Hypo	None	Yes	ON SC		BS	BS SC	BS	BS		
50	PP-MS	Yes	46	59	Hypo	None	Yes	SC was initial and remained only site of clinical involvement						
193	PP-MS	Yes	38	28	Hypo	IDDM age 35	Yes	Cerebrum	SC					
227	PP-MS	NT	49	Before MS	Hypo	Dermatitis herpetiformis	Yes	Progressive SC						
♂313	PP-MS	Yes	57	57	Hypo	Crohn’s, alopecia areata	No	Progressive SC						
337	PP-MS	Yes	50	48	Hypo	None	Yes	Progressive SC						
384	PP-MS	No	34	25	Hyper	None	Yes	Progressive SC						
614	PP-MS	Yes	48	14	Hypo	None	Not known	Progressive Cb and BS						
207	NMO spectrum disorders (NMOSD)	No	70	70	Hypo	Myasthenia gravis	Yes	SC	SC	Died				
219	NMOSD	Yes	26	9	Hyper	Mild psoriasis	Yes	SC	SC	SC	SC	SC	SC	SC
268	NMOSD	No	29	29	Hypo	ITP; APC Abs	No	ON	SC	SC	SC	SC		
328	NMOSD	NT	25	40	Hypo	None	Yes	ON	ON	ON	ON	SC		

*RR-MS, relapsing-remitting MS; SP-MS, secondary progressive MS; PP-MS, primary progressive MS; RR-NMO, relapsing-remitting NMO; SC, spinal cord; BS, brainstem; ON, optic nerve; Cb, cerebellum; IDDM, insulin-dependent (type 1) diabetes mellitus; ITP, immune thrombocytopenic purpura; APC Abs, anti-phosphatidyl choline antibodies; ANA, anti-nuclear antibodies; ♂, male patient*.

**Table 4:** **Time of onset of CNS demyelinating disease compared to onset of autoimmune thyroid disease (AITD) differs in patients with hypothyroidism vs hyperthyroidism**.

	CNS disease occurred first	AITD occurred first	CNS disease and AITD commenced concurrently
Hypo (*n* = 22)	12	7	3
Hyper (*n* = 15)	5	10	0

Eleven of the 44 patients (25%) had an additional autoimmune disease, most commonly psoriasis or Crohn’s disease. Of the 37 patients where a family history was known, 30 (81.1%) had other family members with an autoimmune disease, most commonly AITD, but also MS, psoriasis, Crohn’s disease, rheumatoid arthritis, and SLE. The personal or family incidence of other autoimmune diseases did not appear to be related to whether the AITD type was hypothyroidism or hyperthyroidism.

In most of the patients, the spinal cord was the most common site of clinical involvement (Table 3), with 68% of the 152 attacks recorded in these patients primarily affecting the spinal cord. Eighteen patients also had attacks involving the optic nerve, and 17 patients had attacks involving the brainstem. In most of the MS cases, there was also clinical and/or magnetic resonance imaging evidence of cerebral lesions, but symptoms from these lesions were generally mild.

### HLA Typing

The HLA types carried by the patients with coexisting CNS demyelinating disease and AITD are shown in Table 5, subgrouped according to the type of thyroid disease (hypo vs hyper) and whether AITD developed prior to or after the CNS disease. Blood was not available for HLA typing from 11 patients (9 with hypothyroidism and 2 with hyperthyroidism). The most commonly found genotype was DRB1*03–DQA1*05–DQB1*02: this genotype has previously been reported to occur commonly in patients with AITD. Interestingly, four of the five patients (80%) who developed MS prior to hyperthyroidism carried the MS-related DRB1*15:01 allele, whereas only one (11.1%) of the nine patients (eight MS and one NMOSD) who developed hyperthyroidism prior to CNS demyelinating disease carried this allele. The HLA-DR genotypic and allelic frequencies for these groups are compared with those of 159 healthy individuals and 277 patients with MS alone (all at the two digit serotyping level) in Table 6. In comparison with the MS group, patients with CNS demyelinating disease and hypothyroidism had an elevated frequency of carriage of HLA-DR3 (significantly different without correction for multiple comparisons). The frequency of carriage of DRB1*15:01 allele among patients with coexisting CNS demyelinating disease and AITD was intermediate between the healthy control group and the MS group, but did not reach statistical significance in comparison with either.

**Table 5:** **HLA types carried by people with CNS demyelinating disease and autoimmune thyroid disease (AITD)**.

	AITD type	ID No.	DRB1^a^	DQA1^a^	DQB1^a^	DPB1^a^
**CNS disease occurred prior to AITD**						
	Hypo	31	03:01, 15:01	05:01, 01:02	02:01, 06:02	03:01, 04:01
	Hypo	43	04:04, 15:01	03:01, 01:02	03, 06:02	02:01, 04:01
	Hypo	50	07:01, 13:02	02:01, 01:02	03:03, 06:04	04:01, 04:01
	Hypo	171	01:01/2, 03:01	01:01, 05:01	05, 02:01/2	04:01, 04:02
	Hypo	291	08, 15:01	04:01, 01:02	04:01/2, 06:02	04:01, 04:01
	Hypo	317	03, 10:01	05:01, 01:05	02, 05	02:01, 04:01
	Hypo	328	15:01, 15:01	01:02, 01:02	06:02, 06:02	ND
	Hypo	332	01:01/2, 03:01	01:01, 05:01	05, 02:01/2	03:01, 04:01
^a^	Hypo	582	01:03, 15	ND	ND	ND
	Hyper	145	04:07, 15:01	03:03, 01:02	030:2, 06:02	03:01, 04:01
	Hyper	182	03, 15:01	05:01, 01:02	02:01/2, 06:02	03:01, 61:01N
^a^	Hyper	493	01:03, 15:01	05:05, 01:02	03:01, 06:02	04:01, 04:02
	Hyper	494	04:02, 15:01	03:01, 01:02	03, 06:02	02:01, 04:01
	Hyper	593	03, 04:02	ND	ND	ND

**CNS disease and AITD commenced at the same time**						
	Hypo	207	03, 03	05:01, 05:01	0201/2, 0201/2	0201, 0202
	Hypo	268	04:01, 15:01	03:02, 01:02	0301, 0602	0401, 0401
	Hypo	313	03:01, 13:03	05:01, 05:05	0201/2, 0301	0101, 0602

**AITD occurred prior to CNS disease**						
	Hypo	193	03:05, 04:02	05:01, 03:01	02:01/2, 03:02	79:01, 79:01
	Hypo	200	07:01, 15:01	02:01, 01:02	02:01/2, 06:02	03:01, 19:01
	Hypo	227	07:01, 07:01	02:01, 02:01	02:01, 03:03/6	04:01, 09:01
	Hypo	261	13:01, 15:01	01:03, 01:02	06:03, 06:02	04:01, 05:01
	Hypo	316	15:01, 15:01	01:02, 01:02	06:02, 06:02	ND
	Hypo	337	15:01, 16:01	01:02, 01:02	06:02, 05	04:01, 10:01
	Hyper	133	01:01/2, 04:01	01:01, 03:01	05, 03:02	03:01, 04:01
	Hyper	219	11, 13:14	01:05, 05:05	05, 03:01	04:01, 04:01
	Hyper	269	01:01/2, 03	01:01, 05:01	05, 02	01:01, 20:01
	Hyper	271	03:01, 07:01	02:01, 05:01	02:01/2, 02:01/2	01:01, 04:01
	Hyper	274	03:01, 08:01	04:01, 05:01	02:01/2, 04:01/2	04:01, 04:01
	Hyper	299	01:03, 04	ND	ND	ND
	Hyper	370	10:01, 13:02	01:05, 01:02	05, 06:04	
	Hyper	384	04:01, 15:01	03:03, 01:02	03:02, 06:02	04:01, 04:02
	Hyper	393	07:01, 15:02	02:01, 01:02	03:01, 06:01	ND

**Commencement of AITD in relation to CNS disease not known**						
	Hypo	374	03, 04:04	05:01, 03:01	02:01/2, 03:02	02:02, 14:01

*^a^Disease commenced after β-IFN treatment; ND = not done*.

**Table 6:** **Frequency of HLA-DRB1 alleles in patients with coexisting CNS demyelinating disease and autoimmune thyroid disease (AITD) in comparison to healthy individuals and multiple sclerosis (MS) patients without AITD**.

DRB1 allele	Healthy controls (HC) (*n* = 159)		MS alone (*n* = 277)		CNS disease + AITD (*n* = 33)		CNS disease + hyperthyroidism (*n* = 14)		CNS disease + hypothyroidism (*n* = 19)	
	Genotypic (%)	Allelic (%)	Genotypic (%)	Allelic (%)	Genotypic (%)	Allelic (%)	Genotypic (%)	Allelic (%)	Genotypic (%)	Allelic (%)
01	28 (17.6)	28 (8.8)	39 (14.1)	39 (7.0)	7 (21.2)	7 (10.6)	4 (28.6)	4 (14.3)	3 (15.8)	3 (7.9)
03	35 (22.0)	36 (11.3)	60 (21.7)	69 (12.5)	13 (39.4)*	14 (21.2)*	5 (35.7)	5 (17.8)	8 (42.1)*	9 (23.7)*
04	46 (28.9)	49 (15.4)	70 (25.3)	75 (13.5)	10 (30.3)	10 (15.2)	6 (42.9)	6 (21.4)	4 (21.0)	4 (10.5)
07	37 (23.3)	41 (12.9)	44 (15.9)	48 (8.7)	5 (15.2)	6 (9.1)	2 (14.3)	2 (7.1)	3 (15.8)	4 (10.5)
08	12 (7.5)	12 (3.8)	17 (6.1)	17 (3.1)	2 (6.1)	2 (3.0)	1 (7.0)	1 (3.6)	1 (5.3)	1 (2.6)
09	7 (4.4)	7 (2.2)	3 (1.1)	3 (0.5)	0	0	0	0	0	0
10	2 (1.3)	2 (0.6)	1 (0.4)	1 (0.2)	2 (6.1)	2 (3.0)	1 (7.0)	1 (3.6)	1 (5.3)	1 (2.6)
11	25 (15.7)	26 (8.2)	24 (8.7)	24 (4.3)	1 (3.0)	1 (1.5)	1 (7.0)	1 (3.6)	0	0
12	11 (6.9)	11 (3.5)	9 (3.2)	9 (1.6)	0	0	0	0	0	0
13	31 (19.5)	31 (9.7)	47 (17.0)	50 (9.0)	5 (15.2)	5 (7.5)	2 (14.3)	2 (7.1)	3 (15.8)	3 (7.9)
14	16 (10.1)	17 (5.3)	11 (4.0)	12 (2.2)	0	0	0	0	0	0
15	50 (31.4)	56 (17.6)	171 (61.7)***	200 (36.1)***	16 (48.5)	18 (27.3)	6 (42.9)	6 (21.4)	10 (52.6)	12 (31.6)
16	2 (1.3)	2 (0.6)	6 (2.2)	7 (1.3)	1 (3.0)	1 (1.5)	0	0	1 (5.3)	1 (2.6)

*p ≤ 0.05 vs HC or MS alone, before correction for multiple comparisons.

***p < 0.0001 vs HC, after correction for multiple comparisons.

### T Cell Reactivity

Given the pronounced involvement of the spinal cord in patients with coexisting CNS demyelinating disease and AITD, T cell responses against two antigens that have been reported to be present at high levels in the spinal cord compared to other parts of the nervous system, CGRP, and LGR4, were tested. CGRP levels have been reported to be upregulated not only in the spinal cord but also in the diseased thyroid gland. LGR4, which is expressed in the CNS, particularly in the spinal cord, and also in other sites throughout the body, notably the skin and gastrointestinal tract, is a homolog of TSHR, the target of autoantibodies in Graves’ disease. We also assessed T cell reactivity against the immunodominant region of PLP (PLP_184–209_), since we have previously found that T cell responses to PLP are elevated in a significant proportion of patients with MS (2, 33, 34). Sufficient blood was available for T cell assays from 15 CNS disease + hypothyroid patients and 10 CNS disease + hyperthyroidism patients. In addition, 20 patients with MS alone and 20 healthy individuals without known thyroid disease were also tested.

**Figure 1:**
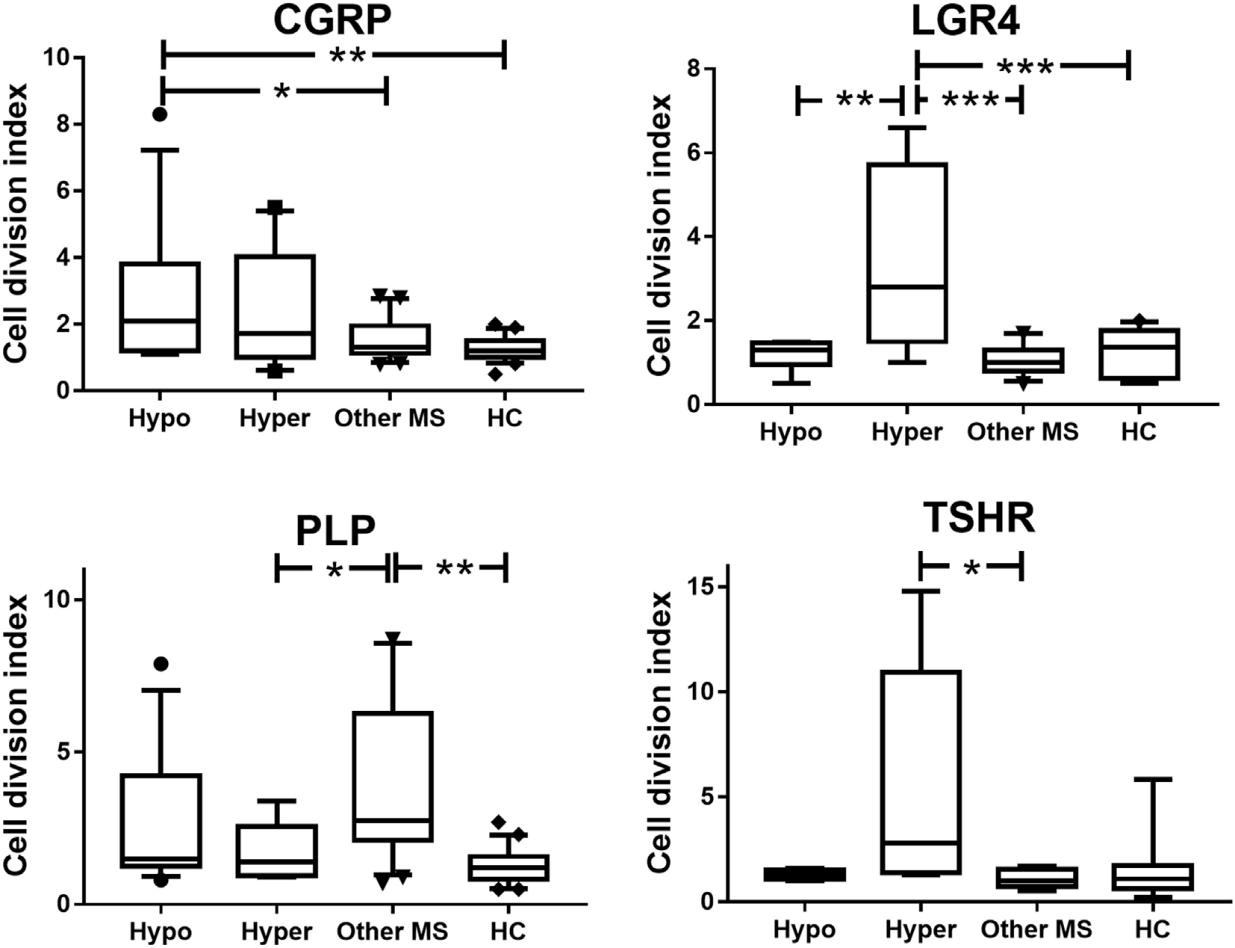
**Proliferative responses to calcitonin gene-related peptide (CGRP), LGR4, thyroid-stimulating hormone receptor (TSHR), and proteolipid protein (PLP) in patients with coexisting CNS demyelinating disease and autoimmune hypothyroidism (Hypo) or hyperthyroidism (Hyper), multiple sclerosis (MS) alone, or healthy controls (HC)**. Because the data are not normally distributed, they were analyzed used the Kruskal–Wallis test (non-parametric equivalent of ANOVA), with Dunn’s multiple comparisons test to allow for multiple comparisons being made in the figure. The box of the box and whisker plots extends from the 25th to the 75th percentiles of the results. The line in the middle of the box is plotted at the median. The whiskers are drawn down to the 10th percentile and up to the 90th. Points below and above the whiskers are drawn as individual points. *p < 0.05 and **p < 0.001, compared to the indicated groups.

Compared to patients with MS alone and to HC, patients with coexisting CNS demyelinating disease and hypothyroidism showed a significant increase in T cell reactivity to CGRP (*p* ≤ 0.03) (Figure 1). T cell reactivity to CGRP was slightly increased in the patients with coexisting CNS demyelinating disease and hyperthyroidism but was not significantly different from that in the group with MS alone or in the HC. In contrast, patients with coexisting CNS demyelinating disease and hyperthyroidism showed significantly increased T cell responses to a panel of three LGR4 peptides, compared to all other groups (*p* ≤ 0.005) (Figure 1). A smaller subgroup (5–8 per group) of those tested for reactivity to LGR4 was also tested for T cell reactivity to the homologous peptides from TSHR. Once again, elevated reactivity to the TSHR peptides was also seen only in the patients with coexisting CNS demyelinating disease and hyperthyroidism (Figure 1); however, owing to the smaller numbers of individuals tested and the variation in the responses of the patients, this did not reach statistical significance.

Compared to the HC, patients with MS without AITD showed significantly elevated T cell reactivity to the PLP_184–209_ region (Figure 1). In patients with coexisting CNS demyelinating disease and AITD, T cell reactivity to PLP_184–209_ was intermediate between that in patients with MS without AITD and that in HC but did not differ significantly from that in either of these groups.

Taken together, these finding show that patients with coexisting CNS demyelinating disease and AITD have increased T cell reactivity directed against antigens present in both the CNS and thyroid or against molecules for which CNS and thyroid homologs exist.

### Antibody Reactivity

Levels of antibodies specific for CGRP were measured in an ELISA assay, using the whole CGRP molecule immobilized on the ELISA plate. Apart from one patient with coexisting MS and hypothyroidism (patient 43), who showed highly elevated serum levels of antibodies against CGRP (>50-fold higher than the level for any other individuals in any group), none of the other patients with coexisting CNS demyelinating disease and AITD showed antibody levels higher than the levels for healthy individuals or other MS patients (data not shown). Interestingly, multiple samples had been collected from patient 43 over 8 years, and testing of all of these samples showed a sustained response to CGRP across the entire 8 years (not shown).

**Figure 2:**
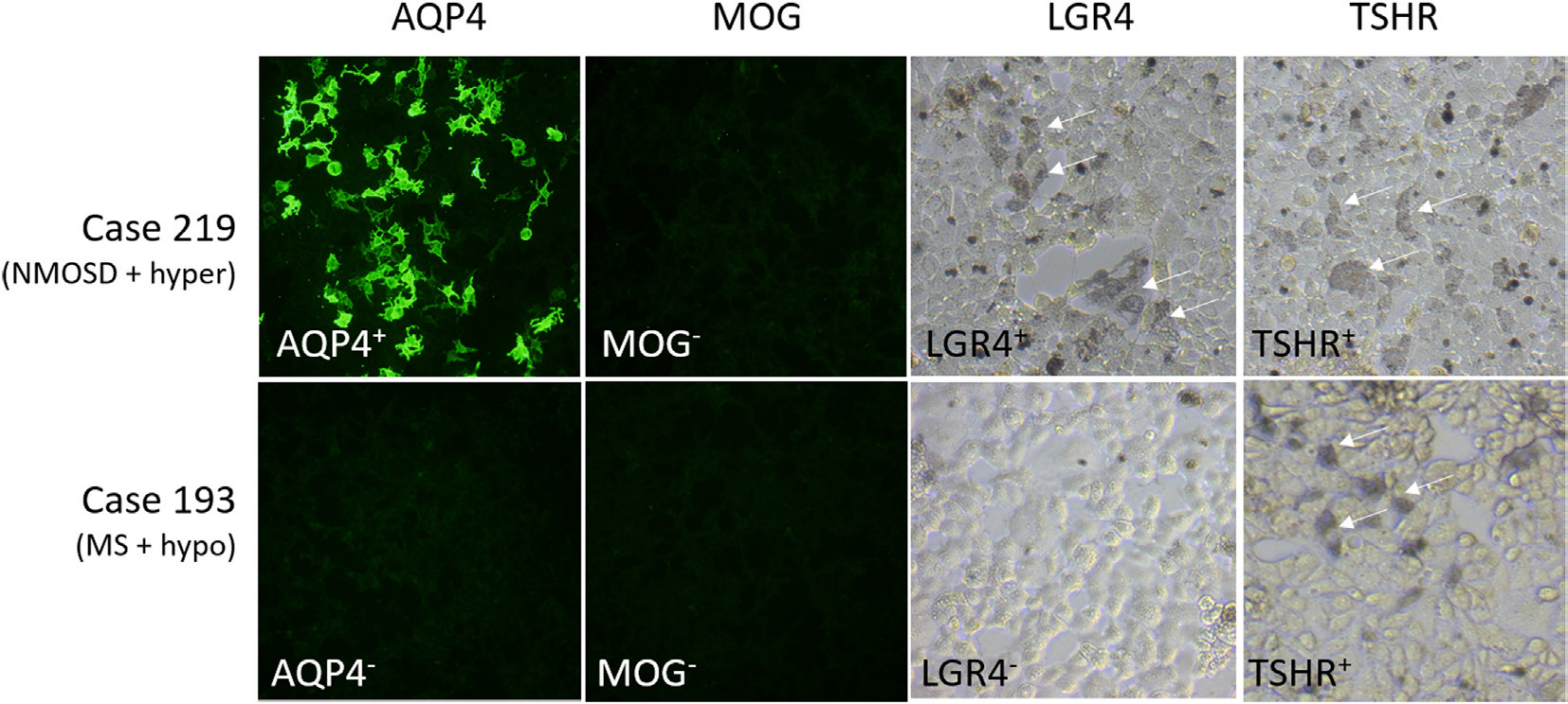
**Representative images of staining of cells expressing aquaporin 4 (AQP4), myelin oligodendrocyte glycoprotein (MOG), LGR4, or thyroid-stimulating hormone receptor (TSHR)**. Results for two cases, #219 [NMO spectrum disorders (NMOSD) patient with hyperthyroidism who is positive for antibodies against AQP4, LGR4, and TSHR] and #193 [multiple sclerosis (MS) patient with hypothyroidism who is positive for antibodies against TSHR, but not the other proteins], are shown. Human antibody labeling of AQP4- and MOG-transfected cells was detected with FITC-labeled anti-human IgG, so that positive cells fluoresced green. Human antibody labeling of LGR4- or TSHR-transduced cells was detected by HRP-labeled anti-human IgG + A + M, followed by nickel-enhanced DAB staining, so that positive cells are labeled by a blue/gray precipitate. Arrows indicate positively stained cells.

Other antibodies were measured using cell-based assays. Results are shown in Table 7, with representative images in Figure 2. Only patients with NMOSD had anti-AQP4 antibodies. None of the patients tested in this study had detectable anti-MOG antibodies using this commercial assay. Labeling of LGR4-transduced cells occurred in 1 of the 4 NMOSD + AITD patients (patient with hyperthyroidism), and in 10 of 30 patients with MS + AITD. In the latter group, 8 of the 10 patients with anti-LGR4 antibodies had hyperthyroidism, consistent with our finding of increased T cell reactivity to LGR4s in patients with concomitant hyperthyroidism and CNS demyelinating disease. Similarly, antibodies specific for TSHR were found mainly in patients with hyperthyroidism (9 of a total of 13 positives). Not all patients who were positive for TSHR antibody also reacted against LGR4 and *vice versa*, suggesting specific targeting of each molecule in different patients.

**Table 7:** **Frequency of antibodies against aquaporin 4 (AQP4), myelin oligodendrocyte glycoprotein (MOG), LGR4 and thyroid-stimulating hormone receptor (TSHR)**.

Patient group	Positive staining of cells transfected with			
	AQP4 (%)	MOG (%)	LGR4 (%)	TSHR (%)
NMO spectrum disorders and autoimmune thyroid disease (AITD) (*n* = 4)	4 (100)	0 (0)	1 (25)	2 (50%)
Multiple sclerosis (MS) and AITD (*n* = 30)	0 (0)	0 (0)	10 (43.3)	11 (36.7)
Hypo (*n* = 18)			2 (11.1)	3 (16.7)
Hyper (*n* = 12)			8 (66.7)	8 (66.7)
MS only (*n* = 14)	ND	ND	0 (0)	2 (14.3)

*ND, not determined*.

## Discussion

In this paper, we report that patients with coexisting CNS demyelinating disease and AITD show novel immune reactivity to CGRP and LGR4 which is not found in MS patients who do not have AITD and HC. The patterns of immune reactivity to these molecules differed between patients with autoimmune hypothyroidism and patients with autoimmune hyperthyroidism, suggesting that immune reactivity targeting these molecules might have a role in the development of the different types of AITD. Also of interest was the clinical presentation of patients, with predominant spinal cord involvement in most patients during one or more attacks.

Patients with increased reactivity to CGRP were distributed among both the hypothyroid and hyperthyroid subgroups, but responses were slightly higher in the hypothyroid group. In contrast to the reactivity to LGR4 in the hyperthyroid group, there were no obvious differences in reactivity to CGRP between patients who developed AITD prior to vs after the CNS disease. Within the CNS, CGRP has been reported to be selectively distributed throughout sensory, motor, and autonomic areas of the spinal cord (35), which may partly explain the predominance of spinal cord disease seen in the patients with coexisting AITD and CNS disease. Reactivity against other thyroid antigens, such as thyroglobulin and thyroperoxidase, were not specifically assessed in the current study, as we focused on antigens that were expressed in the spinal cord; however, antibodies against these molecules were measured as part of the clinical workup of these patients, but there were no significant correlations between the antibody levels and reactivity to CGRP or LGR4 (data not shown).

The most interesting group of patients was those with autoimmune hyperthyroidism preceding the onset of CNS demyelinating disease. They carried HLA molecules different from those generally associated with MS or NMOSD and had significantly elevated T cell and antibody reactivity to LGR4. TSHR and LGR4 both belong to the LGR family, but whereas TSHR belong to the Group A LGR family, which have 7–9 leucine-rich repeats, LGR4 belongs to the Group B family, which have 17 leucine-rich repeats (27). Overall, there is only about 20% identity and 45% similarity between the two molecules, but there are several parts of the molecules, particular in their shared seven transmembrane domain, where the regions of identity and similarity are higher. Studies in patients with Graves’ disease have shown a variety of T cell epitopes in TSHR, with none appearing to be dominant (36). The three peptides from TSHR, and the three corresponding peptides of LGR4 used in this study, which were chosen based on their predicted extracellular location and on their predicted ability to bind to HLA molecules commonly found in MS, only represent a small selection of possible T cell epitopes. It would be useful in the future to undertake a larger T cell study utilizing overlapping peptides across the whole of the similar parts of these molecules. However, even with the limited number of peptides used, we could still see significantly elevated responses to LGR4 in the subgroup of patients with coexisting AITD and CNS demyelinating disease. We postulate that, in these patients, CNS disease might be a direct consequence of immunological cross-reactivity between LGR4 and TSHR, with disease spreading from the thyroid to the CNS.

There are many case reports in the literature of CNS disorders, including myelopathy, myelitis, and ataxia occurring together with thyroid disease (13, 37–41). In many cases, it has been reported that the CNS symptoms can be reversed by normalizing the levels of thyroid hormones, suggesting that the CNS symptoms were caused directly by the altered thyroid hormone levels. However, there are some reports where such therapy has not reversed the CNS disease, but rather the CNS syndrome has remained and progressed (37, 42, 43). In one such study, where persistent cerebellar ataxia was associated with elevated levels of autoantibodies against thyroglobulin and thyroperoxidase, the authors concluded that the most likely cause of the cerebellar degeneration in the patients was autoimmune attack (43). The results of the current study lend further support to the idea that autoreactivity against antigens present in the thyroid could spread to the CNS.

The idea of intra-CNS epitope spreading in animal models of MS and in MS itself is well established (44, 45), although we are not aware of reports where disease spreads from the CNS to another organ. However, another example of where cross-reactivity between related antigens might play a role in spreading of autoimmune disease from a peripheral organ to the CNS (or *vice versa*) is in patients with coexisting type 1 diabetes (T1D) and stiff person syndrome (SPS). In both T1D and SPS, a major target of autoimmune attack is glutamic acid decarboxylase (GAD). GAD exists in two isoforms of differing molecular weight and encoded by separate genes, GAD65 and GAD67, and while both isoforms are present in the CNS, only GAD65 is present in the pancreas. Autoantibodies and T cells from patients with T1D appear to target different epitopes of GAD65 when compared to patients with SPS, and generally the response to antigens in SPS appears to be broader than in T1D, with a larger number of epitopes of both GAD65 and GAD67 being recognized (46, 47).

It is of interest to note that development of AITD has been reported to follow treatment with several of the drugs commonly used in MS, including IFN-β and anti-CD52 antibody (alemtuzumab/Campath/Lemtrada). IFN-β has been reported to enhance the production of the B cell activating factor (BAFF), which could potentially enhance antibody-mediated autoimmune disease. In contrast, anti-CD52 antibody has been suggested to lead to development of other autoimmune disease through the depletion of regulatory T cells. The reason(s) why these treatments results predominantly in AITD is as yet uncertain. Our results suggest that there could be cross-reactivity between antigens in the CNS and the thyroid, and since AITD is likely to be predominantly an autoantibody-mediated disease, enhancement of the autoantibody response or removal of regulatory cells that normally keep the action of these autoantibodies under control could result in the emergence of AITD.

Some studies (48–50) but not others (10, 51) have shown an increased occurrence of other autoimmune diseases in patients with MS and their first-degree relatives, and NMOSD has also been linked to coexistence of other autoimmune diseases in addition to AITD (52). Irrespective of whether or not the overall incidence of other autoimmune diseases is higher or the same as the general population, it is of interest why some patients appear to be so susceptible to multiple autoimmune diseases. It is generally considered that patients who develop multiple autoimmune diseases carry HLA types that predispose them to development of certain combinations of autoimmune diseases, and this would almost certainly be important in determining such potential. However, at present, our knowledge of the specific antigens involved and their HLA restriction is insufficient to predict combinations of autoimmune diseases with any certainty. It is notable that out of all the potential autoimmune diseases that could occur, a fairly restricted number of diseases were reported in both the personal and family histories of patients with coexisting CNS demyelinating disease and AITD, primarily thyroid disease, MS, diseases affecting the skin (including psoriasis, alopecia areata, and dematitis herpetiformis), and disease affecting the gastrointestinal tract (Crohn’s disease, celiac disease). This specific combination of diseases is of interest, as both CGRP and LGR4 are strongly expressed not only in the thyroid and the spinal cord but also in the gastrointestinal tract and in the skin. The number of patients in the current study is too small to determine whether elevated reactivity to CGRP and/or LGR4 definitely correlates with the development of other autoimmune diseases affecting the gastrointestinal tract or skin, but this would be an interesting study for the future. It is also of interest to note that one of the NMOSD patients in our study also had myasthenia gravis, since acetylcholinesterase, one of the targets of autoantibodies in myasthenia gravis, has an extensive degree of homology with thyroglobulin (53). Since myasthenia gravis co-occurs with NMOSD at a relatively high frequency, it would be of interest to determine if patients with both of these diseases were more likely to develop AITD than other NMOSD patients.

The reason for the coexistence of CNS demyelinating disease and AITD almost exclusively in females (43 of the 44 patients in this study were female) is unclear. Most studies report a higher incidence in females than males for MS (typically ~3:1 female: male, although in the south-east part of Queensland where this study was conducted the ratio appears to be higher at ~6:1) (54, 55), NMOSD (2–5.5:1) (20) and AITD (~5:1) (56), and it may just be that the risk associated with getting CNS disease and AITD together represents a fairly straightforward multiplicative relationship between the risk of getting each disease alone. Alternatively, there could be differences in regulation/expression of these antigens in female vs male CNS tissue, dimorphism in the imprinting of the genes encoding these molecules (57), or other mechanisms that remain to be elucidated.

The further study of patients with coexisting AITD and CNS demyelinating disease may help to identify new antigenic targets within the CNS and to explain previous observations of linkages between altered thyroid metabolism and subsequent CNS disease.

## Ethics Statement

This study was carried out in accordance with the recommendations of the NHMRC National Statement on Ethical Conduct in Human Research. All subjects gave written informed consent in accordance with the Declaration of Helsinki. The protocol was approved by the Royal Brisbane and Women’s Hospital Human Research Ethics Committee, The University of Queensland Medical Research Ethics Committee and the Griffith University Human Research Ethics Committee.

## Author Contributions

JG and MP designed the study. MP and SB recruited patients, assessed patients included in the study, and edited the manuscript. JG undertook the laboratory-based research and wrote the manuscript.

## Conflict of Interest Statement

The authors declare that the research was conducted in the absence of any commercial or financial relationships that could be construed as a potential conflict of interest.
